# Galanin Receptors and Ligands

**DOI:** 10.3389/fendo.2012.00146

**Published:** 2012-12-07

**Authors:** Kristin E. B. Webling, Johan Runesson, Tamas Bartfai, Ülo Langel

**Affiliations:** ^1^Department of Neurochemistry, Arrhenius Laboratories for Natural Science, Stockholm UniversityStockholm, Sweden; ^2^Molecular and Integrative Neurosciences Department, The Scripps Research InstituteLa Jolla, CA, USA; ^3^Institute of Technology, University of TartuTartu, Estonia

**Keywords:** galanin, galanin-like peptide, GMAP, alarin, epilepsy

## Abstract

The neuropeptide galanin was first discovered 30 years ago. Today, the galanin family consists of galanin, galanin-like peptide (GALP), galanin-message associated peptide (GMAP), and alarin and this family has been shown to be involved in a wide variety of biological and pathological functions. The effect is mediated through three GPCR subtypes, GalR1-3. The limited number of specific ligands to the galanin receptor subtypes has hindered the understanding of the individual effects of each receptor subtype. This review aims to summarize the current data of the importance of the galanin receptor subtypes and receptor subtype specific agonists and antagonists and their involvement in different biological and pathological functions.

## The Galanin Family

Since the discovery of galanin 30 years ago, several bioactive peptides have been reported to be part of the galanin family. The discovery of galanin was followed by the characterization of a second peptide originating from the same prepropeptide as galanin, the galanin message associated peptide (GMAP). Furthermore, a third peptide, GALP, was identified with capacity to bind to the galanin receptor subtypes, GalR1-3, followed by the characterization of a splice variant of GALP named alarin.

### Galanin

Galanin was discovered among several other bioactive peptides with C-terminal α-amide motif, using a new method by Professor Viktor Mutt and colleagues at Karolinska Institute, Stockholm (Tatemoto et al., 1983; Hökfelt, 2005; Lang et al., 2007). The 29 amino acid long peptide (30 amino acids in humans) was named galanin after its N-terminal glycine and its C-terminal alanine. The N-terminal end of galanin is crucial for its biological activity and the first 15 amino acids are conserved in all species (the tuna fish being the exception; Kakuyama et al., 1997). Interestingly, the C-terminal region (residues 17–29) varies among species and it lacks receptor affinity (Table 2), which is also true for N-terminal fragments shorter than galanin (1–11) (Land et al., 1991b). The C-terminus is believed to primarily serve as a protector against proteolytic attacks (Land et al., 1991a; Bedecs et al., 1995). In a membrane-mimicking environment, galanin adopts a horseshoe-like shape, where the N-terminus is organized in an α-helical conformation, followed by a β-bend around the proline in position 13 and a more uncertain configuration of the C-terminal region (Wennerberg et al., 1990; Morris et al., 1995, Öhman et al., 1998).

Galanin has been ascribed a large range of different functions. To accomplish these, the galanin gene has a highly plastic expression pattern, which has been portrayed numerous times in the literature. Galanin was early shown to be induced by estrogens (Vrontakis et al., 1987, 1989; Kaplan et al., 1988), and later, three copies of estrogen responsive element, ERE, were identified in the promoter region of the human galanin gene (Kofler et al., 1995). Thereafter, the galanin expression has also been shown to be up-regulated by the leukemia inhibitory factor (LIF; Corness et al., 1996; Sun and Zigmond, 1996), and down-regulated by the nerve growth factor (NGF; Verge et al., 1995).

Galanin is widely expressed in the central and peripheral nervous system as well as in the endocrine system and co-exists with a number of classical neurotransmitters, including acetyl choline, serotonin, glutamate, GABA, noradrenalin, and dopamine (Melander et al., 1986; Hökfelt et al., 1987; Xu et al., 1998; Liu et al., 2003). Galanin also co-exists with other neuropeptides like enkephalin, NPY, substance P, vasopressin, calcitonin gene-regulated peptide, and gonadotropin-releasing hormone (Rökaeus and Carlquist, 1988; Merchenthaler et al., 1990; Zhang et al., 1993a,b, 1995).

An extensive up-regulation of galanin was seen during development of sensory and motor systems (Gabriel et al., 1989; Xu et al., 1996) and after nerve injury, both in PNS and CNS (Hökfelt et al., 1987) and also, an extensive up-regulation in the basal forebrain of patients with Alzheimer’s disease (AD; Chan-Palay, 1988a,b). Epileptic seizures have been shown to rapidly deplete galanin (Mazarati et al., 1998).

Galanin has also been shown to be expressed in keratinocytes, eccrine sweat glands and around blood vessels (Kofler et al., 2004). Furthermore, galanin has been proposed to be expressed in macrophages of the dermis (reviewed in Bauer et al., 2010).

### Galanin message associated peptide

There are very few studies regarding the localization, function, and pharmacological potential of GMAP. It was early shown that the sequence of GMAP displays a much greater divergence between species than galanin (Lundkvist et al., 1995). Immunohistochemistry has shown that GMAP distribution generally parallels that of galanin (Hökfelt et al., 1992) although heterologous distribution was observed in certain areas. Xu et al. (1995a,b) showed that GMAP has a pharmacological action in spinal nociceptive transmission in rat spinal cord (Andell-Jonsson et al., 1997; Hao et al., 1999). GMAP has also been assigned anti-microbial activities and hypothesized to be part of the innate immune system, since it suppresses *Candida albicans* growth and the budded-to-hyphal-form transition of *C. albicans* (Rauch et al., 2007) (Table 1). Recently, in an extended study, it was shown that GMAP could significantly reduce growth in six out of seven *Candida* strains (Holub et al., 2011).

**Table 1:** **A short summary of the involvement of the galanin family in different physiological and pathological functions**.

Agonist	Antagonist
**GALANIN RECEPTOR 1**	
Reduces acetylcholine release in cardiac neurons (Potter and Smith-White, 2005)	Anxiolytic and antidepressant (Kuteeva et al., 2008)
Antinocicepive effects for neuropathic pain (Liu et al., 2001)	Improving memory and learning (Wrenn et al., 2004; Bailey et al., 2007)
Treatment for diseases of altered extrinsic afferent signaling around that gastrointestinal tract (Page et al., 2007)	
Anticonvulsant, reduces initiation of seizures (Mazarati et al., 2004b; Bulaj et al., 2008)	
**GALANIN RECEPTOR 2**	
Neuroprotective effects against Alzheimer’s disease (Pirondi et al., 2010)	
Anxiolytic and antidepressant (Kuteeva et al., 2008)	
Anticonvulsant by reduction of severity of seizures (Mazarati et al., 2004a; Robertson et al., 2010)	
**GALANIN RECEPTOR 3**	
Inhibit oxytocin secretion (Radács et al., 2010)	Anxiolytic and antidepressant (Swanson et al., 2005; Kuteeva et al., 2008; Ash et al., 2011)
	Reduced alcohol consumption (Ash et al., 2011)
	Reduces severity of acute pancreatitis (Barreto et al., 2011)
**EXOGENOUS GALANIN**	
Increases heart rate, induces tachycardia and a weak vasodepressor response (Narváez et al., 2000)	
Impaired performance in memory tests (Wrenn et al., 2004; Bailey et al., 2007)	
Increases alcohol intake (Schneider et al., 2007)	
Inhibit the secretion of vasopressin and oxycotin (Ciosek and Cisowska, 2003; Izdebska and Ciosek, 2010; Radács et al., 2010)	
Anticonvulsant effects (Mazarati et al., 1992, 1998, 2000, 2004b; Chepurnov et al., 1998; Lu et al., 2010; Robertson et al., 2010,)	
Anti-nociceptive (Xu et al., 2012)	
Neuroprotective effects against Alzheimer’s disease (Pirondi et al., 2010) Anxiolytic- and antidepressant (Kuteeva et al., 2008)	
**EXOGENOUS GALP**	
In rats: an acute increase (30–60 min) of food intake, followed by reduction in food intake (Lawrence, 2009), increased sexual behavior in male rats (Fraley et al., 2004)	
In mice: decreased food intake (Lawrence, 2009), an acute decrease in body temperature followed by an increase in body temperature (Man and Lawrence, 2008a)	
Inhibition of male sexual behavior in mice (Kauffman et al., 2005)	
**EXOGENOUS ALARIN**	
In male mice: Increase of acute food intake, acute increase of body weight, increased LH levels, decrease of neurogenic inflammation, no change in body temperature (Fraley et al., 2012)	
Anti-edema and vasoconstrictive effects (Santic et al., 2007)	
Increases LH levels in mice and rats (Boughton et al., 2010; van Der Kolk et al., 2010; Fraley et al., 2012)	
**EXOGENOUS GMAP**	
Anti-microbial activities (Rauch et al., 2007; Holub et al., 2011)	
Facilitation of the flexor reflex, decrease of spinal cord blood flow (Xu et al., 1995a)	

### Galanin-like peptide

Ohtaki et al. (1999) characterized a third peptide, isolated from porcine hypothalamus, that was recognized to induce GTP-binding to a membrane preparation of GalR2-transfected cells. They named this new peptide galanin-like peptide, or GALP. Porcine GALP was shown to act as an agonist in a GTPγS binding assay and to have a preferential binding (20 times) toward GalR2 (Ohtaki et al., 1999). A later study using human GALP showed that GALP interacts with GalR3 with three times preferential selectivity as compared to GalR2 (Lang et al., 2005) (Table 2).

**Table 2:** **Affinities of galanin, GALP, GMAP, and alarin, as well as fragments of galanin and GALP, for the three galanin receptor subtypes, determined as K_i_**.

Ligand	*K*_i_ (nM)			Reference
	GalR1	GalR2	GalR3	
Rat galanin(1–29)	1.0	1.5	1.5	Wang et al. (1997b)
	0.3 (h)	1.6 (h)	12 (h)	Borowsky et al. (1998)
	0.9 (h)	1.2 (h)	7.4 (h)	Lu et al. (2005b)
Human galanin(1–30)	0.4 (h)	2.3 (h)	69 (h)	Borowsky et al. (1998)
Porcine galanin(1–29)	0.23 (h)	0.95 (h)	9.8 (h)	Borowsky et al. (1998)
Galanin(1–16)	4.8	5.7	50	Wang et al. (1997b)
Galanin(2–29)	85	1.9	12	Wang et al. (1997b)
Galanin(3–29)	>1000	>1000	>1000	Wang et al. (1997b)
Galanin(2–11)	>5000 (h)	88	271	Lu et al. (2005a)
	879^a^ (h)	1.8^a^	–	Liu et al. (2001)
Porcine GALP	4.3	0.24	–	Ohtaki et al. (1999)
Human GALP	77^a^ (h)	28^a^ (h)	10^a^ (h)	Lang et al. (2005)
Human GALP(1–32)	129^a^ (h)	69^a^ (h)	–	Lang et al. (2005)
Human GALP(3–32)	33^a^ (h)	15^a^ (h)	–	Lang et al. (2005)
Rat GALP	45^a^	18.7^a^	1530^a^ (h)	Boughton et al. (2010)
Alarin	>1000	>1000	>1000000	Boughton et al. (2010)
GMAP(1–41)	–	>840	–	Wang et al. (1997a)
	–	–	>1000	Wang et al. (1997b)
GMAP(44–59)	–	>1000	>1000	Wang et al. (1997b)

*Displacement is performed on the rat galanin receptor unless indicated otherwise. (h) human; ^a^presented as IC_50_ values; – not determined*.

The amino acid sequence of GALP-(9–21) is identical to that of galanin (1–13).

Galanin-like peptide distribution in the CNS appears to be rather restricted, disparate to the much broader expression pattern seen for galanin. Cells identified to produce GALP mRNA and protein have only been found in the hypothalamic arcuate nucleus (ARC), the median eminence and infundibular stalk, and the posterior pituitary when studied in the rat, mouse, and macaque (Juréus et al., 2000, 2001; Kerr et al., 2000; Larm and Gundlach, 2000; Takatsu et al., 2001; Cunningham et al., 2002; Fujiwara et al., 2002). GALP-immunoreactive (IR) fibers were shown to project to several regions of the forebrain (Takatsu et al., 2001).

Galanin-like peptide has also been shown to be expressed by specialized glia-like cells known as pituicytes in the neuronal lobe of dehydrated and salt loaded rats, where the expression is strongly regulated by osmotic stimuli (Shen et al., 2001; Fujiwara et al., 2002; Saito et al., 2003; Shen and Gundlach, 2004). Furthermore, studies show that the GALP gene expression, especially in the pituicytes, is induced by both acute and chronic inflammatory stimuli (Saito et al., 2003, 2005). Central administration of GALP increases IL-1α and IL-1β and it has been suggested that IL-1 mediates both the anorectic and febrile actions of GALP (Man and Lawrence, 2008b).

Intracerebroventricular (i.c.v.) injection of GALP profoundly stimulates male sex behaviors in rat (Fraley et al., 2004), seemingly independent of the testosterone milieu (Stoyanovitch et al., 2005) (Table 1). Interestingly, the opposite is seen in mice were GALP instead inhibits male sex behavior (Kauffman et al., 2005). Recently, Taylor et al. (2009) presented evidence supporting the hypothesis that this effect of GALP depends upon hypothalamic dopamine input to the medial preoptic area (mPOA).

Several studies have proposed that GALP does not solely interact with the three known galanin receptor subtypes (Man and Lawrence, 2008a). Krasnow et al. (2004) reported that GALP injection affect food intake and body weight in a similar manner in both GalR1-KO and GalR2-KO mice compared to wild type littermates. Furthermore, to somewhat exclude the possibility that this effect was mediated through GalR3, the authors showed that the GALP fragment, GALP (1–21), failed to mimic the effect of full length GALP (Krasnow et al., 2004).

### Alarin

The newest member of the galanin peptide family, alarin, a 25 amino acid long peptide named after its N-terminal alanine and its C-terminal serine originating as a splice variant of the GALP mRNA (Santic et al., 2006). The alarin peptide has been isolated from murine brain, thymus, skin (Santic et al., 2007), human neuroblastic tumors, and human skin (Santic et al., 2006, 2007) and has no detectable affinity toward either of the three galanin receptor subtypes (Boughton et al., 2010) (Table 2). Recently, two publications characterized in more detail the alarin-LI in the murine brain (van Der Kolk et al., 2010; Eberhard et al., 2012). Alarin-LI has a much broader expression pattern than GALP and was found in such diverse areas as the accessory olfactory bulb, different nucleus in the hypothalamus, within the locus coeruleus (LC) and locus subcoeruleus of the midbrain.

When first discovered, alarin was ascribed vasoconstrictive and anti-edema activities (Santic et al., 2007) (Table 1). Contradictory to the effect of GALP, alarin has neither an effect on body temperature nor an effect on male sex behaviors in rodents (van Der Kolk et al., 2010; Fraley et al., 2012). Recently, it was shown that alarin stimulates acute food intake and some studies have reported a significant increase in body weight after 24 h, although other studies were unable to confirm this (Boughton et al., 2010; van Der Kolk et al., 2010; Fraley et al., 2012). Central injection of alarin elicit a gonadotrophin-releasing hormone (GnRH)-mediated increase in leutizing hormone (LH)-levels in both rats and mice (Boughton et al., 2010; van Der Kolk et al., 2010; Fraley et al., 2012).

## Galanin Receptor Subtypes

All three galanin receptor subtypes are members of the GPCR superfamily but the subtypes have substantial differences in sites of expression as well as their functional coupling and subsequent signaling activities. These differences between the receptor subtypes contributes to the diversity of possible physiological effects and the plausible pharmacological relevance of targeting the galanin family (Table 1).

### Galanin receptor type 1

The first known galanin receptor, galanin receptor type 1 (GalR1), was isolated from the Bowes human melanoma cell line (Habert-Ortoli et al., 1994) and subsequently rat (Burgevin et al., 1995; Parker et al., 1995) and mouse (Jacoby et al., 1997; Wang et al., 1997c) receptor was cloned.

The human GalR1 gene contains three exons and the hGalR1 gene translates into a 349 amino acid long protein (Jacoby et al., 1997). The homology between species is rather high, as 93% of the residues in rat GalR1 are identical to those of human GalR1 (Jacoby et al., 1997). The expression of GalR1, but neither GalR2 nor GalR3, is regulated by cyclic adenosine monophosphate (cAMP) through the transcription factor CREB (cAMP regulatory element binding protein; Zachariou et al., 2001; Hawes et al., 2005). The GalR1 expression does not fluctuate during development (Branchek et al., 2000; Burazin et al., 2000).

GalR1 mRNA was initially identified by northern blot to be found in the fetal brain and small intestinal tissues (Habert-Ortoli et al., 1994). It has, thereafter, been identified by reverse transcript polymerase chain reaction (RT-PCR) in the gastrointestinal tract (Lorimer and Benya, 1996). However, a later study identified the GalR1 expression to be exclusively in the central and peripheral nervous system (Waters and Krause, 2000), where it was detected in hippocampus, hypothalamus, amygdala, thalamus, cortex, brainstem (medulla oblongata), spinal cord, and dorsal root ganglia (DRG; Gustafson et al., 1996; Waters and Krause, 2000), even if broader central and peripheral tissue distribution has also been reported (Sullivan et al., 1997).

Activation of GalR1 results in a pertussis toxin (PTX) sensitive inhibition of adenylate cyclase (AC) through interaction with Gα_i_/α_o_ types of G-proteins (Habert-Ortoli et al., 1994; Parker et al., 1995; Wang et al., 1997c) which leads to opening of GIRK channels. Activation of GalR1 can also stimulate a mitogen associated protein kinase (MAPK) activity, through a PKC-independent mechanism, consistent with that the mediator is the βγ-subunit of Gα_i_ (Wang et al., 1998).

### Galanin receptor type 2

The second galanin receptor type (GalR2) was identified in rat hypothalamus, spinal cord, and DRG (Fathi et al., 1997; Howard et al., 1997; Smith et al., 1997; Ahmad and Dray, 2004) and subsequently in mouse spleen (Pang et al., 1998) as well as from various human tissues (Bloomquist et al., 1998; Borowsky et al., 1998). The human GalR2 has rather high sequence identity to rat GalR2 (92%), although there is one notable difference; the 15 amino acid extension of the C-terminal end in human GalR2 (Kolakowskim et al., 1998; Waters and Krause, 2000).

GalR2 is able to activate the stimulatory pathway of Gα_q/11_ class of G-proteins, i.e., PTX-insensitive. This triggers PLC activity and intracellular phosphoinositol turnover, mediating the release of Ca^2+^ into the cytoplasm from intracellular stores and opening Ca^2+^-dependent channels (Smith et al., 1997; Kolakowskim et al., 1998; Wang et al., 1998). GalR2 is also able to activate MAPK through a PKC and Gα_o_ class of G-proteins dependent mechanism (Wang et al., 1998). This may in turn lead to the downstream PI3K-dependent phosphorylation of Protein Kinase B (PKB) leading to suppression of caspase-3 and caspase-9 activity (Ding et al., 2006; Elliott-Hunt et al., 2007). GalR2 activation may also inhibit forskolin stimulated cAMP production in a PTX-sensitive manner, suggesting the activation of Gα_i_/α_o_ types of G-proteins (Fathi et al., 1997; Wang et al., 1997a). Consequently, both GalR1 and GalR2 activation can inhibit CREB (Badie-Mahdavi et al., 2005).

GalR2 is expressed in a wider pattern, compared to GalR1, as it is found in several peripheral tissues including the pituitary gland, gastrointestinal tract, skeletal muscle, heart, kidney, uterus, ovary, and testis as well as in regions in the CNS (Smith et al., 1997; Bloomquist et al., 1998; Waters and Krause, 2000). In the brain, the highest levels of GalR2 are detected in hypothalamus, dentate gyrus, amygdala, piriform cortex, and mammillary nuclei (Mitchell et al., 1999; O’Donnell et al., 1999; Waters and Krause, 2000).

Interestingly, GalR2 expression levels vary during the development of the rat brain with a broader distribution with a peak in expression before postnatal day 7, particularly in cortex and thalamus, and much reduced levels after postnatal day 14 (Burazin et al., 2000).

### Galanin receptor type 3

Galanin receptor type 3 (GalR3) was first isolated from rat hypothalamic cDNA libraries (Wang et al., 1997b) and later from human cDNA (Kolakowskim et al., 1998; Smith et al., 1998). The 368 amino acid long hGalR3 shares 36% amino acids identity with hGalR1 and 58% with hGalR2 and approximately 90% with rGalR3 (Kolakowskim et al., 1998).

The distribution pattern of GalR3 is somewhat unclear but it is assumed that this receptor has a more restricted expression pattern in relation to the other two receptors. Transcript levels is most prominent in the hypothalamus (Wang et al., 1997b; Smith et al., 1998; Mennicken et al., 2002) although, some studies report a wider distribution of GalR3 throughout central and peripheral tissues (Kolakowskim et al., 1998; Waters and Krause, 2000).

Signaling properties of GalR3 are still ill-defined. Activation of GalR3 expressed in *Xenopus* oocytes or *Xenopus* melanophores leads to the activation of Gα_i_/α_o_ type of G-proteins inhibiting AC which results in the opening of GIRK channels (Kolakowskim et al., 1998; Smith et al., 1998).

## Peptide Ligands for the Galanin Receptors

Endogenous galanin has high affinity for all three galanin receptors (Wang et al., 1997b). The N-terminal part of galanin is crucial for receptor interaction and the galanin fragment galanin (1–16) retains the high affinity of its parental peptide. When galanin (1–16) underwent an L-alanine scan and subsequent testing on rat hypothalamus membranes, Gly^1^, Trp^2^, Asn^5^, Tyr^9^, and Gly^12^ were identified as pharmacophores (Land et al., 1991b). A later study, which tested an identical set of peptides on separated GalR1 and GalR2 membranes, identified Trp^2^, Tyr^9^, and Leu^10^ as pharmacophores on both receptor subtypes (Carpenter et al., 1999).

Several N-terminal truncated galanin fragments have been shown to have a preference for GalR2 (Wang et al., 1997b; Liu et al., 2001), in concurrence with the fact that Gly^1^ is of great importance for ligand binding to GalR1. Further truncation, with as little as two amino acids, leads to a complete loss of receptor affinity to all receptor subtypes (Wang et al., 1997a).

Liu et al. (2001) published the galanin fragment galanin (2–11) as a GalR2 selective agonist, although they did not test it on GalR3 (Table 3). Later publication has unfortunately shown that it has similar affinity toward GalR3 (Lu et al., 2005a), without testing receptor signaling, even so, it has been used extensively as a non-GalR1 agonist. Lundström and colleagues showed that Trp^2^, Asn^5^, Gly^8^, Tyr^9^, and Leu^10^ were identified as crucial for interactions with GalR2 by performing Ala-scan on the peptide (Lundström et al., 2005a).

**Table 3:** **Published ligands and their affinities for the galanin receptor subtypes**.

Peptide	*K*_i_ (nM)			*K*_i_ (GalR1)/ *K*_i_ (GalR2)	*K*_i_ (GalR3)/ *K*_i_ (GalR2)	Reference
	GalR1	GalR2	GalR3	
M1151	98.6	28.9	874	3.4	30	Saar et al. (2011)
M1152	2370	36.4	656	65	18	Saar et al. (2011)
M1153	1890	4.98	230	380	46	Saar et al. (2011)
M1145	587	6.55	497	90	76	Runesson et al. (2009)
M15	0.65	1.0	1.0	0.65	1	Smith et al. (1998)
M35	0.11 (h)	2.0 (h)	–	0.055	–	Borowsky et al. (1998)
	0.325	3.24	2.09	0.1	0.64	Smith et al. (1998)
	4.8	8.2	4.7	0.58	0.57	Lu et al. (2005b)
M40	2.4 (h)	4.1 (h)	–	0.58	–	Borowsky et al. (1998)
	6.76	3.55	79.4	1.9	22.3	Smith et al. (1998)
	1.8	5.1	63	0.35	12.3	Lu et al. (2005b)
M617	0.23 (h)	5.7 (h)	–	0.04	–	Lundström et al. (2005b)
	–	–	49 (h)	–	–	Sollenberg Eriksson et al. (2010)
M871	420 (h)	13 (h)	–	32.3	–	Sollenberg Eriksson et al. (2006)
	–	–	>10000 (h)	–	–	Sollenberg Eriksson et al. (2010)
Gal-B2	3.5 (h)	51.5 (h)	–	0.019	–	Bulaj et al. (2008)
[N-Me,des-Sar]Gal-B2	364 (h)	20 (h)	-	18.2	–	Robertson et al. (2010)
Gal2–11	>5000 (h)	88	271	56.8	3.08	Lu et al. (2005a)

*The sequences and structures of the ligands are listed in Table 4*.

*Displacement was performed on the rat galanin receptor unless indicated otherwise. (h) human; – not determined*.

**Table 4:** **The sequences for the galanin family peptides along with the discussed analogs**.

Name	Sequence	
**GALANINFAMILY**		
Rat galanin(1–29)	GWTLNSAGYLLGPHAIDNHRSFSDKHGLT-amide	
Human galanin(1–30)	GWTLNSAGYLLGPHAVGNHRSFSDKNGLTS	
Porcine galanin(1–29)	GWTLNSAGYLLGPHAIDNHRSFHDKYGLA-amide	
Galanin(1–16)	GWTLNSAGYLLGPHAI-amide (rat/porcine)	
	GWTLNSAGYLLGPHAV-amide (human)	
Rat Galanin(2–29)	WTLNSAGYLLGPHAIDNHRSFSDKHGLT-amide	
Rat Galanin(3–29)	TLNSAGYLLGPHAIDNHRSFSDKHGLT-amide	
Galanin(2–11)	WTNLSAGYLL-amide	
Porcine GALP	APVHRGRGGWTLNSAGYLLGPVLHPPSRAEGGGKGKTALGILDWKAIDGLPYPQSQLAS	
Human GALP	APAHRGRGGWTLNSAGYLLGPVLHLPQMGDQDGKRETALEILDLWKAIDGLPYSHPPQPS	
Human GALP(1–32)	APAHRGRGGWTLNSAGYLLGPVLHLPQMGDQD	
Human GALP(3–32)	AHRGRGGWTLNSAGYLLGPVLHLPQMGDQD	
Rat GALP	APAHRGRGGWTLNSAGYLLGPVLHPPSRAEGGGKGKTALGILDLWKAIDGLPYPQSQLAS	
Alarin	APAHRSSTFPKWVTKTERGRQPLRS (human)	
	APAHRSSPFPPRPTRAGRETQLLRS (mouse)	
GMAP(1–41)	ELPLEVEEGRLGSVAVPLPESNIVRTIMEFLSFLHLKEAGA (rat)	
GMAP(44–59)	SLGIPLATSSEDLEQS (rat)	
**PEPTIDE LIGANDS**		
M1151	GWTLNSAGYLLGPK(ε-NH-C(O)Glu)-amide	
M1152	WTLNSAGYLLGPK(ε-NH-C(O)Glu)-amide	
M1153	RGRGNWTLNSAGYLLGPK(ε-NH-C(O)Glu)-amide	
M1145	RGRGNWTLNSAGYLLGPVLPPPALALA-amide	
M15	GWTLNSAGYLLGPQQFFGLM-amide	
M35	GWTLNSAGYLLGPPPGFSPFR-amide	
M40	GWTLNSAGYLLGPPPALALA-amide	
M617	GWTLNSAGYLLGPQPGFSPFR-amide	
M871	WTLNSAGYLLGPEHPPPALALA-amide	
Gal-B2	(Sar)WTLNSAGYLLGPKKK(palmitoyl)K-amide	
[N-Me,des-Sar]Gal-B2	(N-Me)WTLNSAGYLLGPKKK(palmitoyl)K-amide	
**NON-PEPTIDE LIGANDS**		
Galnon	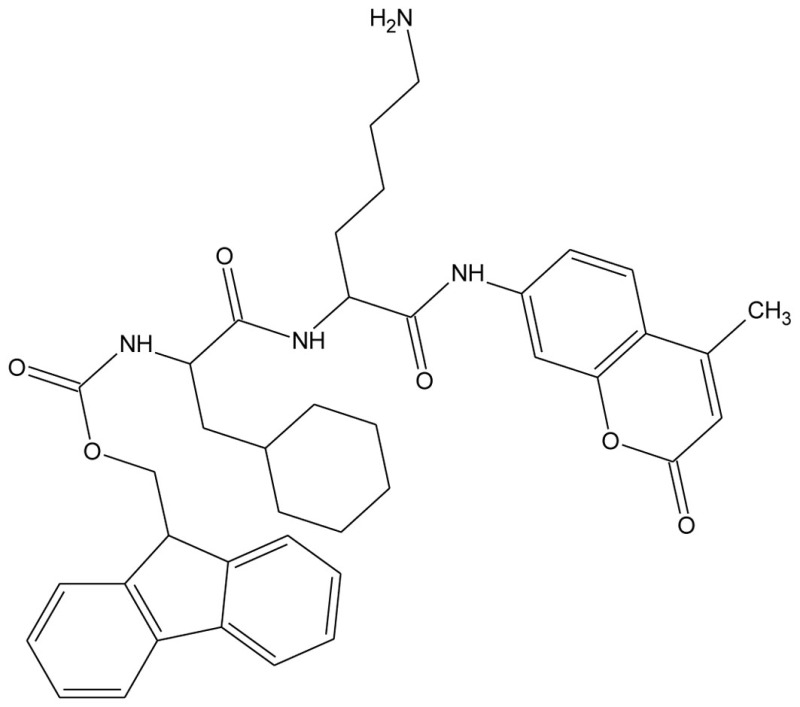	GalR1–3 agonist
Galmic	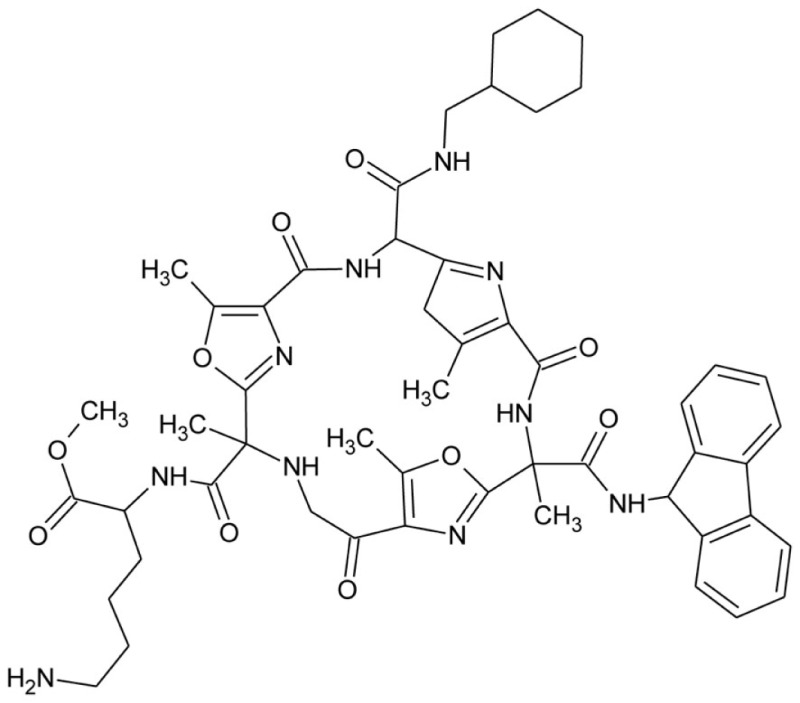	GalR1 agonist
Sch 202596	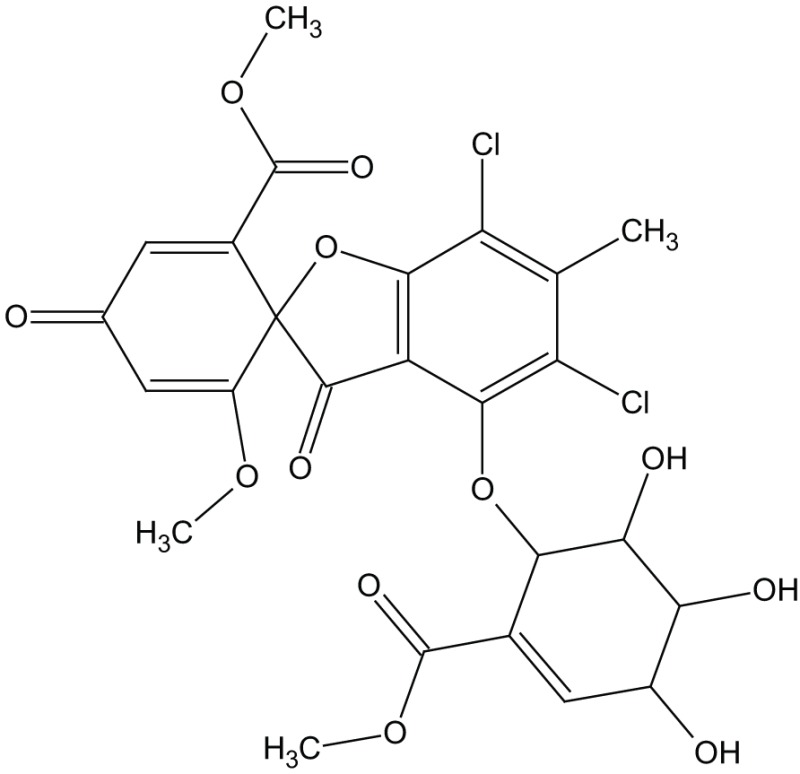	GalR1 antagonist
Dithiepine-1,1,4,4,-tetroxide	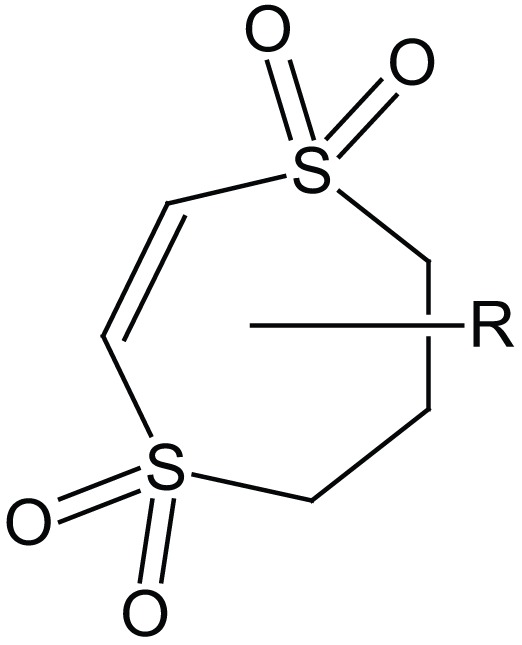	
SNAP 37889	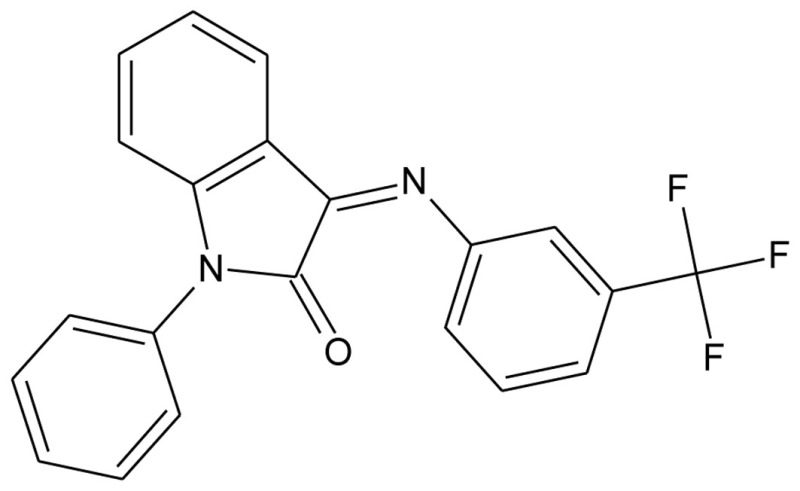	GalR3 antagonist
SNAP 398299	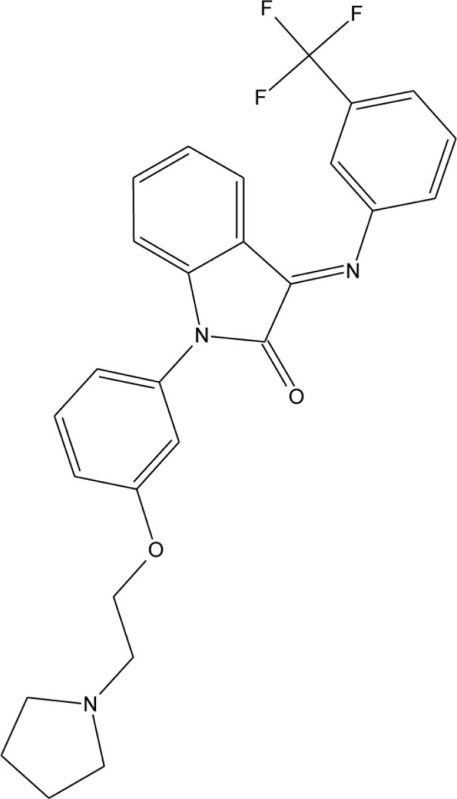	GalR3 antagonist
GalR3ant	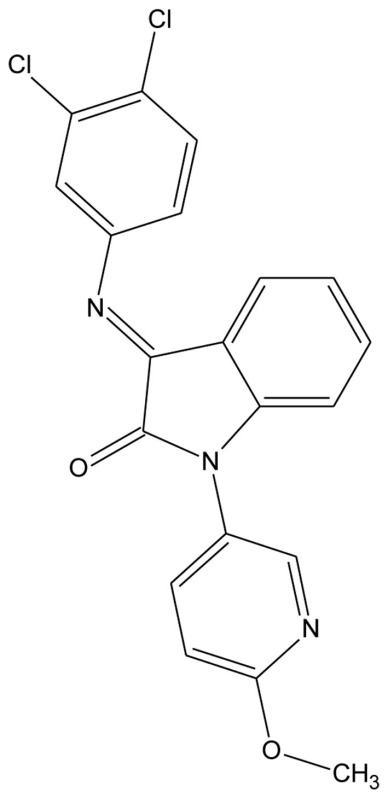	GalR3 antagonist

*Sar, sarcosine*.

The interaction between the galanin receptor subtypes and GALP has received less attention. GALP, isolated from porcine tissues, was original published as a GalR2 preferring ligand, with a 20 times difference in affinity between GalR1 and GalR2 (Ohtaki et al., 1999). Later it was shown, using human GALP, that GALP also interacts with GalR3. In this study GALP was ascribed a GalR3 preferential selectivity (3 times differences; Lang et al., 2005). Recently, Boughton et al. (2010) showed a more than 10 times preferential binding toward GalR3 for the rat GALP (Table 2).

Several chimeric ligands have been synthesized, conjugating galanin (1–13) to other bioactive molecules, yielding M15 (also called galantide; Bartfai et al., 1991), M32 (Wiesenfeld-Hallin et al., 1992b), M35 (Wiesenfeld-Hallin et al., 1992b, Ögren et al., 1992, Kask et al., 1995), C7 (Langel et al., 1992), and M40 (Langel et al., 1992; Bartfai et al., 1993). Although, they all maintain antagonistic properties *in vivo* at doses between 0.1 and 10 nmol when delivered i.c.v. or intrathecally (i.t.; Parker et al., 1995; Lu et al., 2005b), they all have a partial agonistic nature *in vivo* at doses higher than 10 nmol when delivered i.c.v. or i.t. (Kask et al., 1995; Lu et al., 2005b).

The first introduced chimeric peptide which acts as an antagonist of the galanin receptor family was M15 (Bartfai et al., 1991). Here, the galanin (1–13) fragment, was coupled to a C-terminal fragment in substance P (residue 5–11), reported to have agonistic effect on the substance P receptor. M15 showed an about 10-fold higher affinity than the endogenous galanin to unspecified subtypes of the galanin receptor family in membrane preparations of rat tissues. Later, M35 was synthesized (Ögren et al., 1992) with an improved *in vivo* stability (Wiesenfeld-Hallin et al., 1992b). M15, M32, M35, and M40 have similar affinity as galanin and have been valuable tools in galanin research but are limited by their relative non-specificity toward the different galanin receptors (Ögren et al., 1992) and by their weak interactions with other receptors than the galanin receptors (Wiesenfeld-Hallin et al., 1992a).

M617 resembles the M35 peptide, with the substitution of proline at position 14 to a glutamine, which results in a 25-fold selectivity for GalR1 over GalR2 *in vitro* (Table 3). M617 has thereafter been shown to produce anti-nociceptive effects (Jimenez-Andrade et al., 2006) and to delay the development of seizure in an animal model (Mazarati et al., 2006). The M871 peptide is N-terminally truncated and has two additional amino acid residues compared to the M40 peptide and function as a partial agonist, selective for GalR2 (Sollenberg Eriksson et al., 2006, 2010). M871 has been used in several *in vivo* studies (Jimenez-Andrade et al., 2006; Alier et al., 2007; Kuteeva et al., 2008). Several GalR2 selective agonists have been reported over the years (Pooga et al., 1998; Runesson et al., 2009; Saar et al., 2011). Small changes in the N-terminus of galanin have been associated with lost binding affinity. However, recently analogues with modifications at both N-terminus and C-terminus have been presented, namely M1145 (Runesson et al., 2009) and M1153 (Saar et al., 2011). M1145 was reported as the first specific GalR2 agonist with a 90-fold binding preference for GalR2 over GalR1 and 76-fold preference over GalR3 (Runesson et al., 2009). The importance of the development of M1145 and M871 and other subtype selective agonists and antagonists can almost not be overestimated and is the key to a successful delineation of galaninergic system and to identify its potential as a therapeutic target.

Recently, several galanin analogs, all modified by introducing several cationic amino acid residues and a palmitoyl moiety was shown to exhibit improved bioavailability after systemic administration (Bulaj et al., 2008; White et al., 2009). One of these, the Gal-B2, with a slight selectivity toward GalR1 (Table 3), was shown to have anticonvulsant effect in several tested animal models (White et al., 2009). In a later study, Bulaj and colleagues modified Gal-B2 to obtain a ligand with an 18 times preferential binding toward GalR2, which displayed similar anticonvulsant activity as the parental peptide (Robertson et al., 2010). Future characterization will probably identify other potential application of Gal-B2 and other systemically active galanin analogs.

## Non-Peptide Ligands

The non-peptide ligand galnon was identified after screening a combinatorial peptidomimetic library (Table 5). It acts as an agonist in functional studies both *in vitro* and *in vivo* (Saar et al., 2002; Bartfai et al., 2004). It has been evaluated in models of anxiety and depression (Rajarao et al., 2007), feeding (Abramov et al., 2004), and pain (Wu et al., 2003). Galmic (Table 5) is a non-peptide agonist with higher affinity for GalR1 compared to GalR2, which under conditions of intrahippocampal administration was 6-fold more potent than galnon in inhibiting self-sustaining status epilepticus (SE), an *in vivo* model for epilepsy (Bartfai et al., 2004; Ceide et al., 2004). Nevertheless, both galnon and galmic potentials are limited by the fact that they have multiple sites of interactions, i.e., D2 dopamine receptors, grehlin and melanocortin receptors, which produce unwanted physiological effects (Florén et al., 2005; Lu et al., 2005b).

**Table 5:** **Affinities of non-peptidergic galanin receptor ligands for the three galanin receptor subtypes, determined as K_i_ on human receptor subtypes**.

Ligand	*K*_i_ (nM)			Reference
	GalR1	GalR2	GalR3	
Galnon	11700	34100	–	Saar et al. (2002)
Galmic	34200	>100000	–	Saar et al. (2002)
Sch 202596	1700	–	–	Chu et al. (1997)
Dithiepine-1,1,4,4-tetroxide	190^a^	>30000^a^	–	Scott et al. (2000)
SNAP 37889	>10000	>10000	17.4	Swanson et al. (2005)
SNAP 398299	>1000	>1000	5.3	Swanson et al. (2005)
GalR3ant	>10000	>10000	15	Barr et al. (2006)

*The structures of the ligands are listed in Table 4*.

*Displacement is performed on the rat galanin receptor unless indicated otherwise*.

*^a^presented as IC_50_ values; – not determined*.

The metabolite Sch 202596 (Table 5), originated from an *Aspergillus* sp. culture found in an abandoned uranium mine in Tuolemene County California, was found to have a modest affinity to GalR1 *in vitro* (Chu et al., 1997). Sch 202596 was characterized as a molecule with a spirocoumaranone skeleton and has only partly been synthesized so far (Katoh et al., 2002). Several 1,4-dithiins and dithiipine-1,1,4,4-tetroxides with binding affinity to GalR1 were identified at the R. W. Johnson Pharmaceutical Institute (Scott et al., 2000). The compound 2,3-dihydro-2-(4-methylphenyl)-1,4-dithiepine-1,1,4,4-tetroxide (Table 5) was shown to be a submicromolar antagonist. It has an IC_50_ of 190 nM for GalR1 and above the highest tested concentration (30 μM) for GalR2. However, its reactive nature and its low solubility makes it unattractive from a therapeutic point of view. Nevertheless, it has been used and evaluated in several studies (Mahoney et al., 2003; Kozoriz et al., 2006).

A series of 3-imonio-2-indolones were identified as specific GalR3 antagonists, with K_i_-values for GalR3 as low as 17 nM and above the tested 10 μM for the other receptors studied (Konkel et al., 2006a). One of these was referred as SNAP37889 (Swanson et al., 2005) (Table 5). One drawback of the above mentioned indolones is the low aqueous solubility (less than 1 μg/ml) which motivated further studies, leading to the identification of a compound with an increased water solubility and selectivity, 1,3-dihydro-1-[3-(2-pyrrolidinylethoxy)phenyl]-3-[[3-(trifluoromethyl)phenyl]imino]-2*H*-indol-2-one, referred as SNAP398299 (Swanson et al., 2005; Konkel et al., 2006b) (Table 5). Another of the synthesized indolones (Table 5) was evaluated *in vivo* by Barr et al. (2006), which together with the other articles and several patent applications (Konkel et al., 2004) indicates that specific GalR3 ligands are in development.

A series of 2,4,6-triaminopyrimidines were recently introduced by The Scripps Research Institute (Sagi et al., 2011). They present both GalR1 and GalR2 selective compounds with K_i_-values starting from 330 nM. Further development of these compounds is likely ongoing and published in due course. Studies from the same institute led to characterization of the first identified allosteric modulator, named CYM2503, for the galanin receptor family, i.e., GalR2 (Lu et al., 2010). CYM2503 failed to displace galanin in binding studies and showed no detectable signaling by itself, but potentiated the effect of galanin when administered simultaneously (Lu et al., 2010).

### Galanin ligands as possible therapeutics for epilepsy

Among the early reported biological effects of galanin were the decreased excitability of myenteric neurons (Tamura et al., 1988) and cardiac ganglia (Konopka et al., 1989). These findings, together with reports that the hippocampus, which is a key structure for the initiation and maintenance of seizures, have a considerable amount of galaninergic innervation (Lu et al., 2005b) draw attention to galanin as a possible anticonvulsant (Mitsukawa et al., 2008).

Mazarati et al. (1992) reported that galanin had an anticonvulsant effect in a picrotoxin-kindled seizure model. Since then, galanin has been shown to up-regulated in several models of SE (in adult rats), i.e., in kainic acid-induced SE (Wilson et al., 2005) and after perforant path stimulation-induced SE (Mazarati et al., 1998). Galanin administrated i.c.v. had anticonvulsant activity in rodents exposed to either PTZ or Li-pilocarpine (Chepurnov et al., 1998; Mazarati et al., 1998, 2000). Similar results were obtained when SE was induced by perforant path stimulation (Mazarati et al., 1998, 2004a).

The galanin receptor subtypes present in the hippocampus have been investigated and both GalR1 and GalR2 are present in relatively high levels (Lu et al., 2005b) with GalR1 mRNA in CA-fields and GalR2-mRNA in the dentate gyrus (Burazin et al., 2000). The involvement of GalR3 in hippocampus is still not well characterized.

GalR1-KO mice displayed a more severe seizure phenotype when SE is induced by either perforant path stimulation or Li-pilocarpine exposure but not when induced by KA exposure compared to WT (Mazarati et al., 2004b). Li-pilocarpine exposure resulted in cell death in CA1, an effect that was elevated in GalR1-KO mice (Mazarati et al., 2004b). Inbred mice with a lower expression of GalR1 has a larger cell loss than wildtype littermates in several hippocampal regions when exposed to KA (Kong et al., 2008; Schauwecker, 2010) without any alteration in seizure parameters. Some studies has also reported that GalR1-KO mice exhibit spontaneous epilepsy (Jacoby et al., 2002; Fetissov et al., 2003; McColl et al., 2006) although other studies could not replicate this phenotype (Mazarati et al., 2004b).

GalR2-KO mice display no difference in seizure susceptibility in two model of SE compared to WT (Gottsch et al., 2005). In contrast to the knockout mice, application of a putative GalR2 specific ligand shorten the SSSE duration and decreased the seizure density and seizure episodes in the perforant path stimulation model, but not the duration of single seizure episodes (Mazarati et al., 2004a). Similar effects were reported after addition peptide nucleic antisense (PNA) oligonucleotide that mediated transient downregulation of GalR2. PNA-treatment resulted in an increase in the severity of SSSE after perforant path stimulation (Mazarati et al., 2004a). Increased damage to hilar interneurons was also seen after PNA-application (Mazarati et al., 2004a).

Acute administration of two systemically active non-selective subtype galanin receptor agonists, galnon, and galmic, has been shown to prevent self-sustained seizure activity (Saar et al., 2002; Bartfai et al., 2004) and penthylenetetrazole (PTZ)-induced seizures (Saar et al., 2002). Galnon has shown to interact with several other receptors (Florén et al., 2005), although the anticonvulsant effect seems to be mediated via GalR1, as pretreatment with a GalR1-specific PNA attenuates its anticonvulsant properties (Saar et al., 2002).

In concordance with this, application of non-selective subtype galanin receptor antagonists has been shown to worsen the severity of SE in several models, i.e., kainic acid-induced seizures (Reiss et al., 2009), hippocampal kindling model (Kokaia et al., 2001), self-sustained SE (SSSE), and PTZ-induced convulsions (Chepurnov et al., 1998; Mazarati et al., 1998, 2000; Saar et al., 2002). A recent study showed that M15, a non-selective subtype galanin receptor antagonist significantly induced cell death in several hippocampal areas although no differences in the latency of onset or duration of severe seizures were seen (Schauwecker, 2010).

Galanin-KO mice have a lower threshold for developing SE after perforant path stimulation or KA exposure compared to WT (Mazarati et al., 2000). Furthermore, Gal-KO mice displayed a neuronal injury in the CA3-region that was not present in WT littermates (Mazarati et al., 2000). In concordance with this, Galanin-OE mice have a higher threshold for SE induced by either perforant path stimulation or PTZ and KA exposure compared to WT (Mazarati et al., 2000). Gal-OE mice have been shown to be less affected during hippocampal kindling, a model for human complex partial epilepsy (Kokaia et al., 2001).

Utilizing a recombinant adeno-associated viral (AAV) system that overexpresses galanin resulted in a dramatic reduction in KA-induced seizure episodes and the total time spent in seizures although no reduction of cell damage was seen (Lin et al., 2003). The same vector delayed the initiation of convulsions at generalized seizure stages and shortened the duration of electrographic after discharges in rats undergoing hippocampal kindling (Kanter-Schlifke et al., 2007). A similar AAV system that overexpresses galanin together with the fibronectin secretory signal sequence succeeded to the attenuation of KA-induced seizures and the neuronal death after KA exposure (Haberman et al., 2003).

A recent study showed that a GalR2 allosteric modulator increased the latency to the first electrographic seizure, decrease the total time in seizure and decreased the mortality in the Li-pilocarpine SE-model (Lu et al., 2010).

Furthermore, acute administration of the systemically active subtype galanin receptor agonist, Gal-B2, with a moderate GalR1 preferential binding, prevents seizures in the 6 Hz mouse model of pharmacoresistant epilepsy (Bulaj et al., 2008). It was later shown to be active also in other seizure and epilepsy models (White et al., 2009). An analog with a moderate GalR2 preferential binding [N-me, des-Sar]Gal-B2, also prevent seizure in the 6 Hz mouse model (Robertson et al., 2010). The authors conclude that these GalR1- and GalR2 preferential analogs (with 15 and 18 times selectivity, respectively) exhibit similar levels of anticonvulsant activity in the 6 Hz mouse model.

In summary, the wide involvement of galanin family peptides in physiological and pathological conditions has drawn attention to this neuropeptide family. Among the earliest areas of interests was the usage of galanin as a possible anticonvulsant.

Due to the three different galanin receptors specific expression in the CNS, several attempts have been made trying to characterize the contribution of each receptor and delineate their effects. Unfortunately, more selective or specific ligands are still needed.

Recent publications of stable peptide ligands have made new administration routes available as well as attract attention from the pharmaceutical industry.

## Conflict of Interest Statement

The authors declare that the research was conducted in the absence of any commercial or financial relationships that could be construed as a potential conflict of interest.
